# Atlas Subluxation Complex, National Upper Cervical Chiropractic Association Intervention, and Dizziness Improvement: A Narrative Review of Historical Perspectives, Literature Synthesis, and a Path for Future Care

**DOI:** 10.7759/cureus.79310

**Published:** 2025-02-19

**Authors:** Tyler Steward

**Affiliations:** 1 Independent Research, Upper Cervical Research Foundation, Hudson, USA

**Keywords:** atlas subluxation complex, chiropractic, dizziness, meniere’s disease, national upper cervical chiropractic association, vertebral subluxation, vertigo

## Abstract

Dizziness is a non-specific and common condition in which the afflicted individual experiences abnormal sensations such as lightheadedness, imbalance, or a false sense of spinning (vertigo). The experience of "dizziness" can result from a wide spectrum of abnormal physiological states, including exhaustion, hypotension, and hypoglycemia, but could also indicate a serious underlying health issue. Since it has many potential generating causes, accurate identification of the underlying etiology of dizziness can present a challenge to clinicians, often resulting in ineffective treatments. We present a hypothesis that atlas subluxation complex (ASC) may comprise an etiological agent of dizziness that can be successfully addressed with National Upper Cervical Chiropractic Association (NUCCA) chiropractic care. In this review, we discuss the pathophysiology of the ASC, introduce the NUCCA chiropractic procedure, and complete a literature review and synthesis. Conceptual evidence, case reports, and theory provide foundational evidence that the ASC may be a contributory factor of dizziness generation and that NUCCA chiropractic corrective care of the ASC may produce favorable dizziness outcomes. However, high-quality studies are lacking. The foundation evidence provides indication that further research via observational studies and randomized controlled trials (RCTs) is warranted.

## Introduction and background

Background

Dizziness is a common condition, affecting over 20% of adults in the United States annually [1,2]. "Dizziness" is a term used to describe many sensations, such as vertigo (a false sense of spinning), lightheadedness (a sense of floating and faintness), and imbalance. Dizziness is one of the top reasons geriatric patients visit their primary care provider [1,2]. Though benign paroxysmal positional vertigo (BPPV) is the most common cause of vertigo and has a resounding cure rate, this is not the case for chronic conditions that cause dizziness [3,4]. Unfortunately, overall, the diagnosis of the generating cause of dizziness is suboptimal, and the treatments are relatively ineffective, leaving about half of patients with long-term disability [4,5]. Dizziness patients don't always fit into the perfect "diagnostic box" either [1]. These staggering facts present the need for better diagnostics, more informed providers, and expanded treatment options. In this narrative review, we present a hypothesis that atlas subluxation complex (ASC) may comprise an etiological agent of dizziness and evaluate the potential role of the National Upper Cervical Chiropractic Association (NUCCA) corrective care of ASC in addressing the condition of dizziness.

Definitions

Due to dizziness being a non-specific diagnosis and used to describe many sensations, along with chiropractic historically having profession-unique nomenclature, this review shall use the following descriptions for its definitions (Table 1). The definitions were compiled from recent PubMed® indexed papers, the published NUCCA standards and protocols consensus, the World Health Organization (WHO), and the Bárány Society’s Consensus papers [6].

**Table 1:** Definitions [6-13] ASC: Atlas subluxation complex; NUCCA: National Upper Cervical Chiropractic Association; CCJ: Craniocervical junction; UCLF: Upper cervical low-force (procedure); UCT: Upper cervical technique; HVLA: High-velocity, low-amplitude; HIO: Hole-in-one

Term	Definition	Comment
Vertigo	The sensation of self-motion when no self-motion is occurring or the sensation of distorted self-motion during an otherwise normal head movement.	No comment
Dizziness	The sensation of disturbed or impaired spatial orientation without a false or distorted sense of motion.	No comment
Vestibulo-visual symptoms	Visual symptoms that usually result from vestibular pathology or the interplay between visual and vestibular systems. These include false sensations of motion or tilting of the visual surround and visual distortion (blur) linked to vestibular (rather than optical) failure.	No comment
Postural symptoms	Balance symptoms related to maintenance of postural stability, occurring only while upright (seated, standing, or walking).	No comment
Lightheadedness	The feeling of impending blackout or faint in the absence of spinning and positional vertigo.	No comment
ASC ©	This term is a neologism intended to denote the far reaching and damaging effects of the subluxated occipital-atlanto-axial area of the cervical spine upon the spinal column and the human organism. It differs in meaning from the commonly used chiropractic term “atlas subluxation” or “atlas-axis subluxation” in that the term "atlas subluxation complex" embraces the demonstrable mechanical and neurological phenomena which, through research, have been found to be associated with the subluxation of the occipital-atlanto-axial spine. Therefore, by definition, the term includes the atlas vertebra in all its planes of misalignment, its positional relationship to the occiput, subjacent vertebrae and pelvis, inclusive of the excursions of these structures into any or all of the bodily orientation planes; and resulting in concomitant detriment to the susceptive neurological components.	NUCCA definition: © Dr Ralph R. Gregory
ASC ©	A structural misalignment of the CCJ resulting in neuropathophysiological changes and bodily dysfunction.	Layman’s simple definition
ASC Syndrome ©	In this term, the word "syndrome" is limited in meaning to include only the observable and measurable signs of an ASC: objective signs. ASC is defined, therefore, as those signs which are always present and measurable in proportion to the intensity of ASC: misalignment factors as shown by X-ray, resulting traction of the neurological component, presence of spastic contracture of the lumbar and pelvic musculature, distortion of the pelvic girdle, displacement of the body’s center of gravity, contractured leg, and deviation of the spinal segments from the vertical axis of the body.	NUCCA definition: © Dr Ralph R. Gregory
Restoration Principle ©	The reduction to normal of the misalignment factors of the ASC. This includes all methods and systems that reduce to or towards normal, the misalignment factors of the ASC. The Restoration Principle, which is based upon specific and acceptable principles of misalignment reduction, therefore is a pre-determined and pre-directed process of correction. In further simplification, “The principle that misaligned vertebra must be maximally restored to normal."	NUCCA definition: © Dr Ralph R. Gregory
Misalignment factors	The misalignment factors are the measurable misalignments of the vertebrae of the spinal column and the positional relationship of the occiput to the spinal column and include the relationship of these structures to the vertical axis of the body and into any and all planes of motion as well as the ratio of magnitude that exists between an excursion into any given plane to that of any other plane of motion.	No comment
CCJ	The junction of the base of the skull and the cervical spine, including the occipital bone, surrounding the foramen magnum (occiput) (C0), C1 (atlas), C2 (axis), and the intervening tendons and ligaments. The specialized articulations between the occipital condyles and the complex ligamentous system link these three structures into one functional unit. This includes neurovascular structures extending from the skull base to C2.	No comment
CCJ misalignment	Improper orthogonal positioning of the skull, atlas, axis, and lower neck in relation to one another. The CCJ misalignment is the structural component of the ASC.	No comment
Vertebral subluxation	Vertebrae that are misaligned relative to the vertical axis in one or more orientation planes resulting in neurological stresses which produce measurable distortion of the spine, pelvis, and contiguous structures.	NUCCA definition
Vertebral subluxation	A lesion or dysfunction in a joint or motion segment in which alignment, movement integrity, and/or physiological function are altered, although contact between joint surfaces remains intact. It is essentially a functional entity, which may influence biomechanical and neural integrity.	WHO definition
UCLF	A calculated and vectored manual force given by the chiropractor to the patient to reduce the misalignment factors; also commonly referred to as the “correction”, or the “adjustment”.	UCLF is not a manipulation. Manipulation occurs when a joint is brought into its paraphysiological joint space with an HVLA force. UCLF has very little excursion and focuses on joint realignment. Manipulation’s primary goal is to restore motion to a fixated joint.
UCT	A chiropractic specialty with an established set of procedures that uses image-guided analysis to measure misalignment factors, addresses the ASC, and uses UCLF as its intervention.	The UCT organizations that belong to the International Chiropractors Association – Council on Upper Cervical Care are as follows: NUCCA, Orthospinology, Atlas Orthogonal, Advanced Orthogonal, Blair, Knee Chest Upper Cervical Society, Evolutionary Percussive Instrument Corrections, Grostic, and HIO

Historical overview

**Figure 1:**
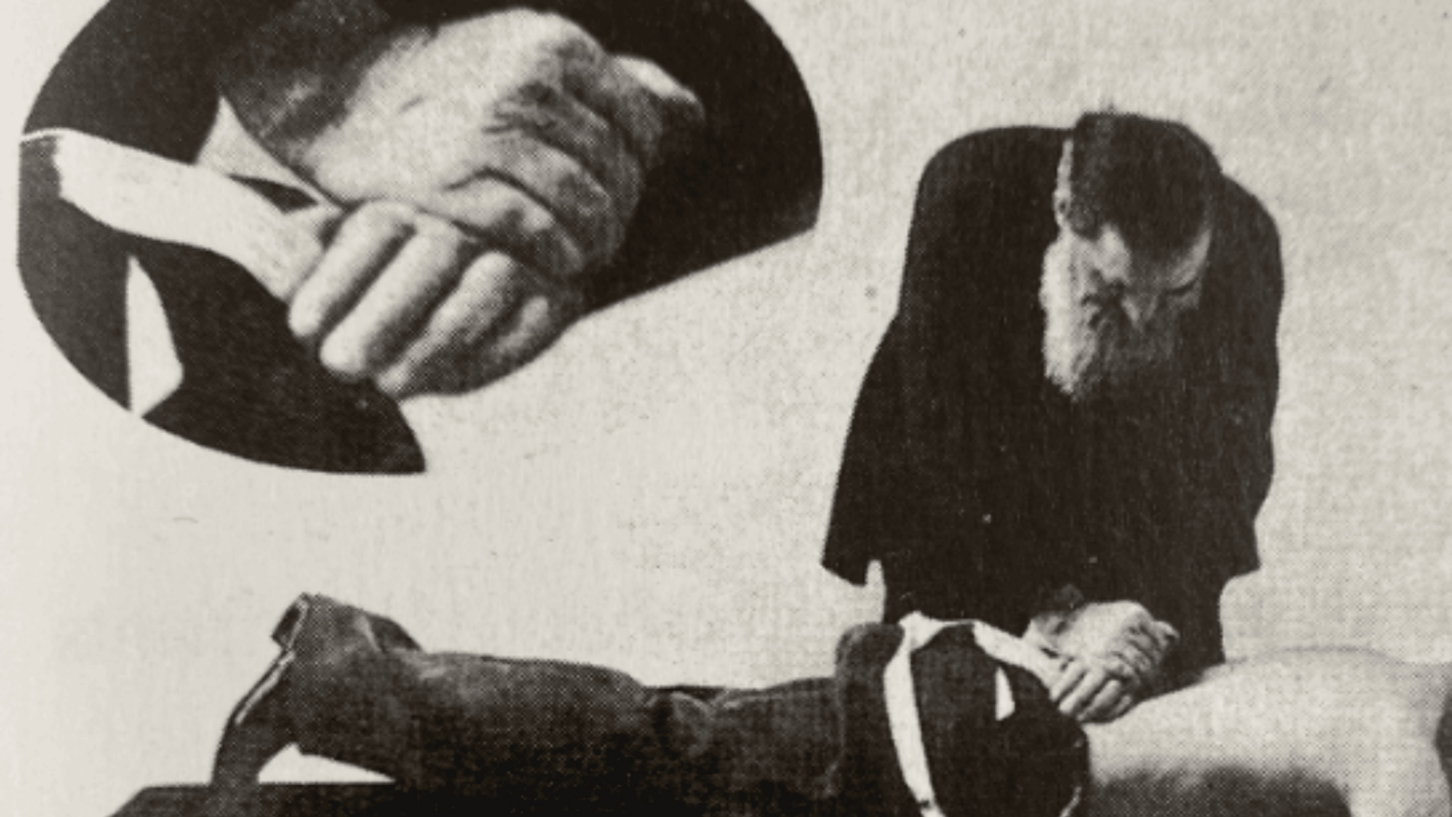
D.D. Palmer showing how the chiropractor places his hands for an adjustment Courtesy of Special Collections and Archives, Palmer College of Chiropractic. Published in *NUCCA Protocols and Perspectives: A Textbook for the National Upper Cervical Chiropractic Association *[16]. Permission was obtained from both sources for use in this article.

Chiropractic care is a separate and distinct healthcare discipline focusing on care of the functional condition of the spine with respect to its influence on the function and adaptability of the nervous system. In 1895, then energy healer D.D. Palmer was attempting to treat a patient with deafness that was acquired after a spinal trauma [14,15]. Palmer’s energetic treatments were ineffective, leading him to try new methods. He noticed the patient had what he perceived to be a spinal bone “out of place.” With ingenuity, Palmer decided to use a high-velocity, low-amplitude (HVLA) force by hand to manually direct the vertebra back into place (Figure 1). The patient reported a complete restoration of hearing after this treatment. Palmer termed this new practice as "chiropractic."

His son, B.J. Palmer, was known as the developer of the chiropractic profession [17]. Soon after the inception of chiropractic, B.J. took an interest in the idea that spinal misalignments were the leading cause of whole-body dysfunction. He named this vertebral abnormality a subluxation [18]. Though the term "subluxation" already existed to describe a minor, or “less than” a dislocation, Palmer rephrased it to mean a misalignment of spinal bones, placing physical pressure on neurological structures, resulting in “nerve interference” and ultimately end-organ disease [18]. Therefore, his definition required two components, the structural misalignment of spinal vertebrae and the resulting nerve interference. His intervention was the chiropractic adjustment, a vectored force by hand used to guide the vertebrae into proper alignment, relieving the nerve interference and restoring bodily function [18]. 

 In 1931, a subset of the profession further focused on the analysis and postural correction of the upper neck region or craniocervical junction (CCJ), where they believed the primary subluxation of the spine existed [19]. Though proper research was almost non-existent at the time, they believed the CCJ region was the primary area of concern due to observation and its proximity to the brainstem. The belief was that subluxation of the CCJ lead to "secondary" subluxations elsewhere in the spine. Therefore, instead of focusing on addressing all of the spinal subluxations, their belief was that correcting the CCJ subluxation would fix the secondary subluxations.

**Figure 2:**
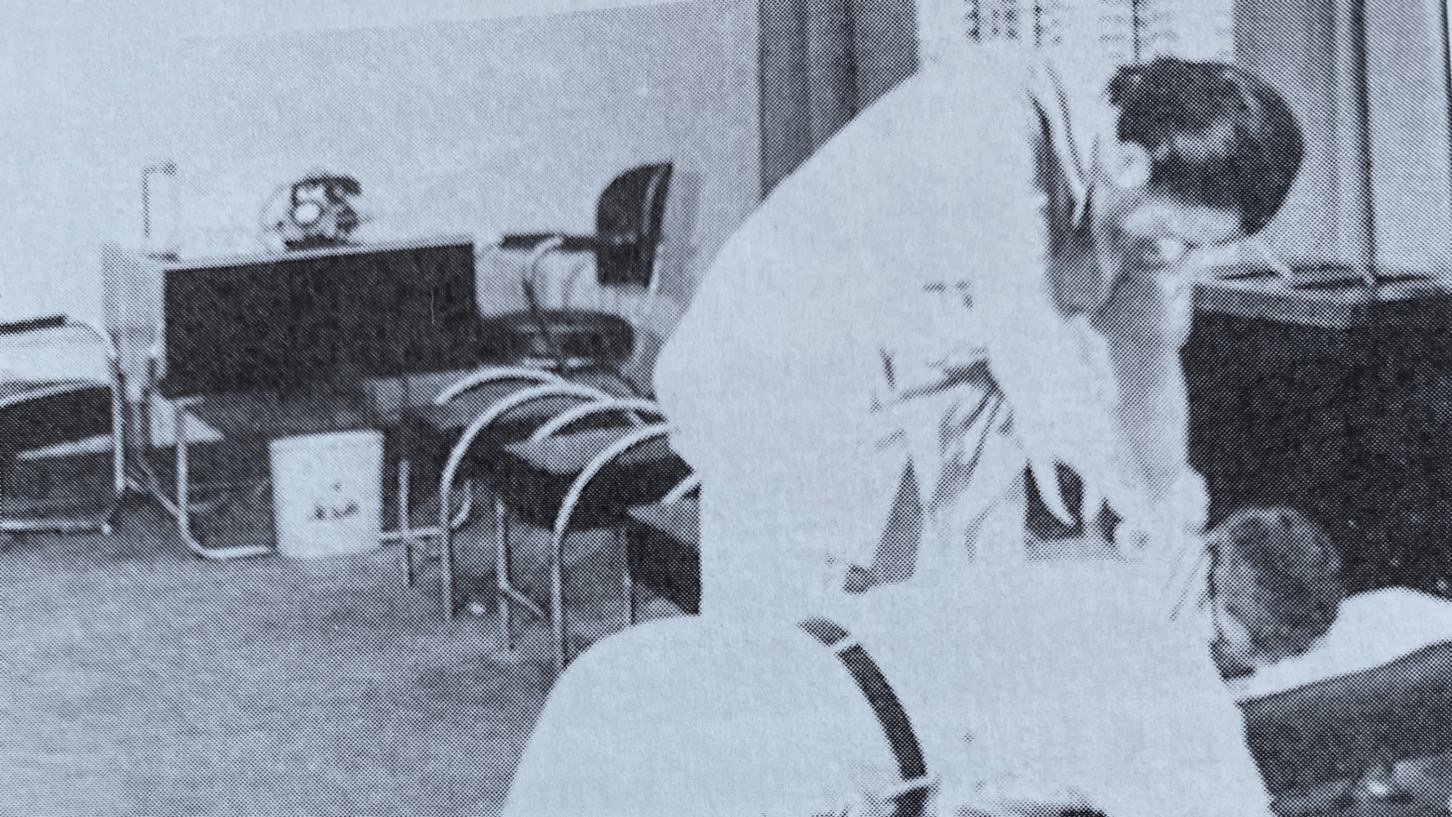
B.J. Palmer showing how the chiropractor places his hands for a HIO adjustment Courtesy of Special Collections and Archives, Palmer College of Chiropractic. Published in *NUCCA Protocols and Perspectives: A Textbook for the National Upper Cervical Chiropractic Association* [16]. Permission was obtained from both sources for use in this article. HIO: Hole-in-one

The hole-in-one (HIO) technique was introduced as the first upper cervical chiropractic method. The method utilized radiographic imaging to objectively measure and assess the alignment of the atlas (C1). The measurements create a two-dimensional vector that the chiropractor would use to improve the alignment by administering an HVLA force using the atlas transverse process as the contact point with the patient lying on their side. The headpiece of the table used a cocking mechanism that allowed it to drop down about two centimeters during the adjustment for added movement. Figure 2 shows Palmer demonstrating how the chiropractor places his hands for an HIO adjustment. 

**Figure 3:**
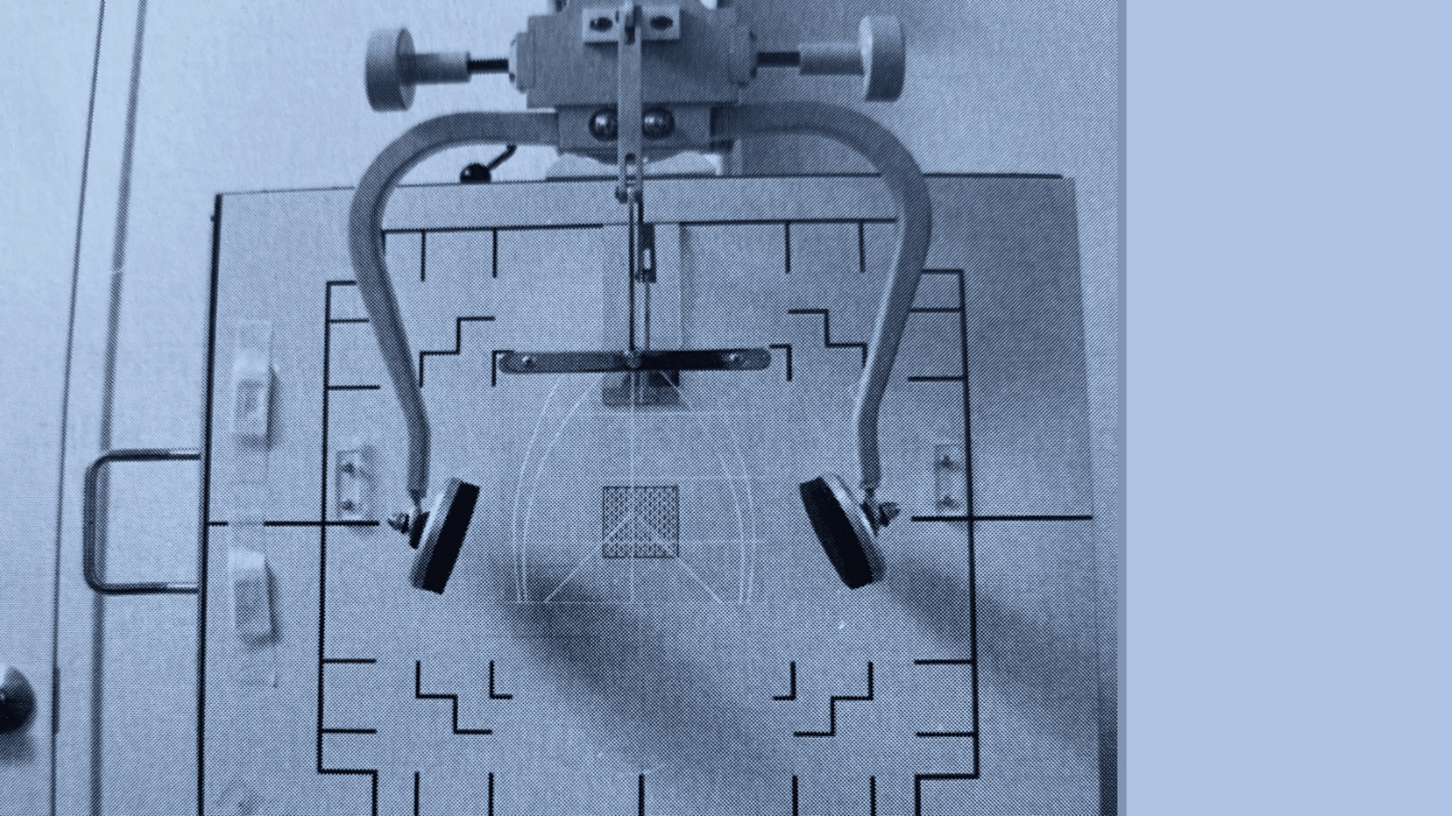
Head clamps used for accurate X-ray positioning Courtesy of and published in* NUCCA Protocols and Perspectives: A Textbook for the National Upper Cervical Chiropractic Association* [16]. Permission was obtained for use in this article.

**Figure 4:**
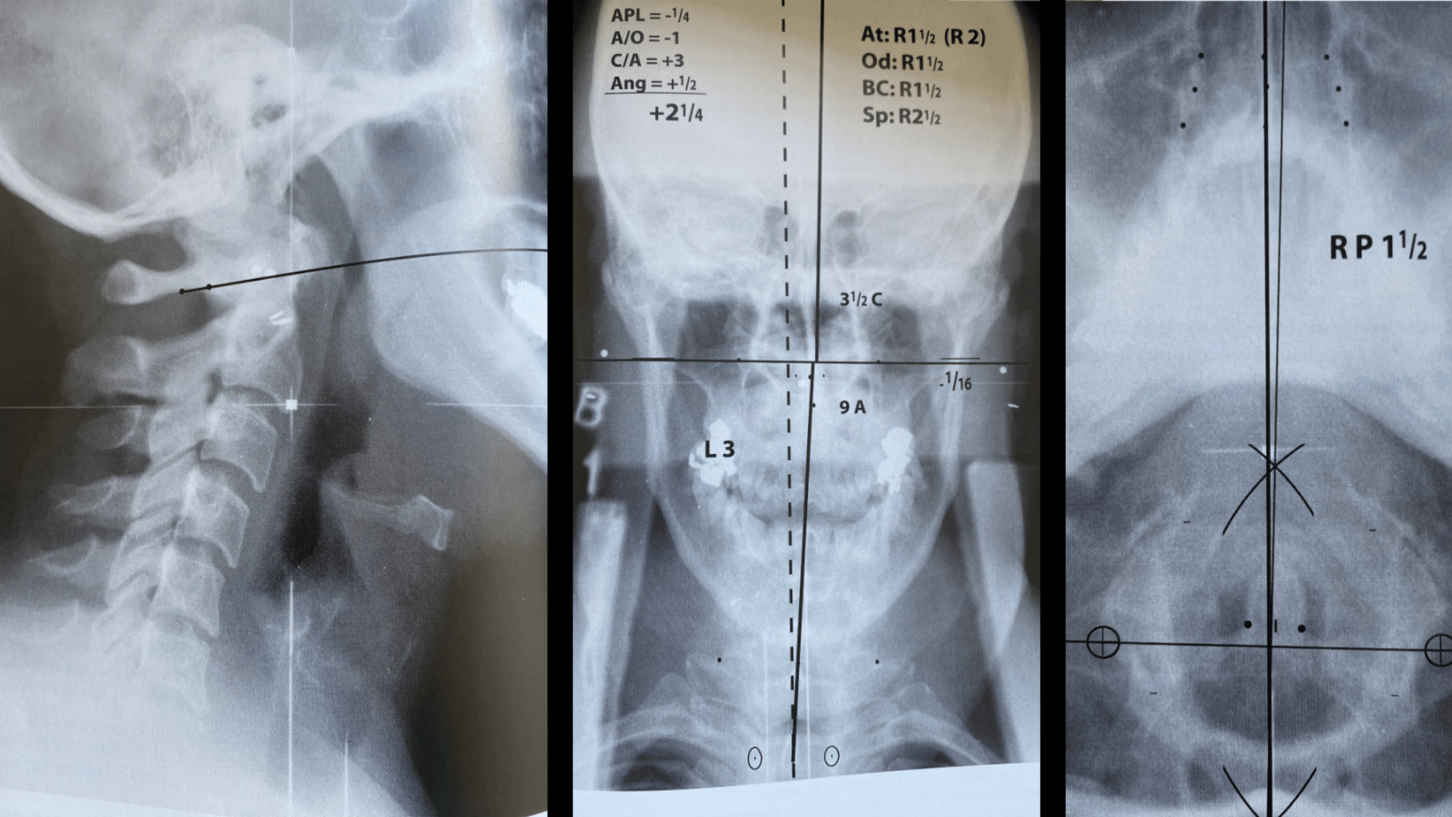
NUCCA radiographs and analysis examples Lateral cervical (left), nasium (middle), and vertex (right) Courtesy of and published in *NUCCA Protocols and Perspectives: A Textbook for the National Upper Cervical Chiropractic Association* [16]. Permission was obtained for use in this article. NUCCA: National Upper Cervical Chiropractic Association

The HIO concept was later expanded upon in 1938 by Dr John Grostic Sr and Dr Ralph R. Gregory, who wished to incorporate metrics to improve inter-practitioner reliability [19]. They introduced the concept of intra-procedural post-adjustment imaging to objectively measure alignment changes after the initial intervention. They created X-ray positioning equipment to reduce radiographic alignment errors (Figure 3). They also expanded upon the analysis to provide a three-dimensional vector for the adjustment [18]. The nasium radiograph view was added for more in-depth analysis (Figure 4). Emphasis was also added on the total orthogonal alignment of the center of the skull, atlas and axis (C1 and C2), and base of the cervical spine [18,19]. The adjustment, developed by Gregory, was updated to be low force and low amplitude, with no movement of the table headpiece. A recent study on inter-examiner reliability of the radiographic analysis using the NUCCA protocol found almost perfect agreement (>96%), without proportional bias [20].

In 1966, Dr Ralph Gregory and colleagues formed the NUCCA organization to further expand the orthogonal chiropractic management of what Gregory referred to as the "atlas subluxation complex" (ASC) [19]. They chose the name to end the era of forming technique protocols around an individual figure as a leader.

**Figure 5:**
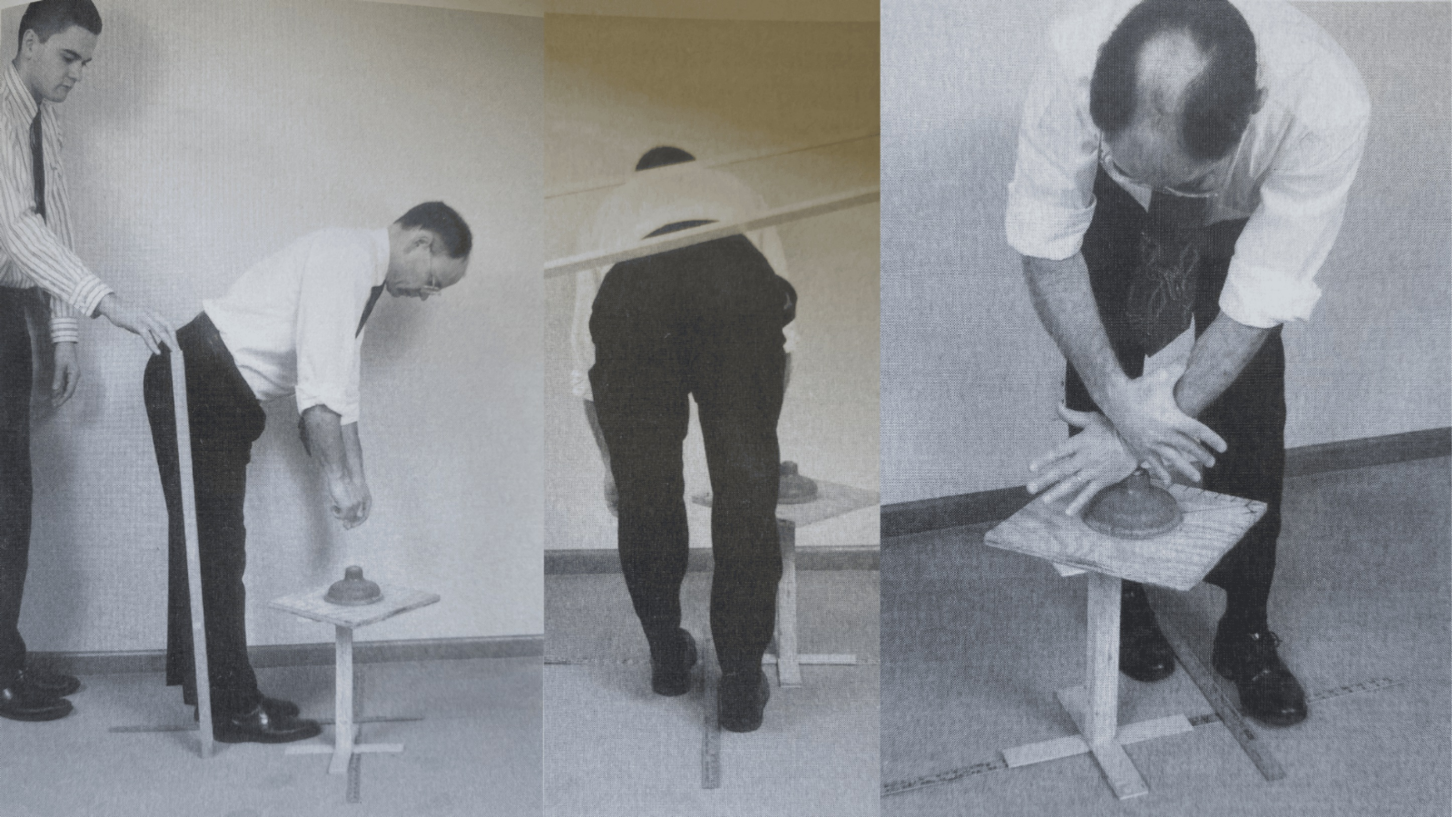
NUCCA intervention setup showing step 2 of phase 1 (left), step 2 of phase 2 (middle), and step 6 of phase 5 (right) as a few examples of the entire process Courtesy of and published in *NUCCA Protocols and Perspectives: A Textbook for the National Upper Cervical Chiropractic Association* [16]. Permission was obtained for use in this article. NUCCA: National Upper Cervical Chiropractic Association

**Figure 6:**
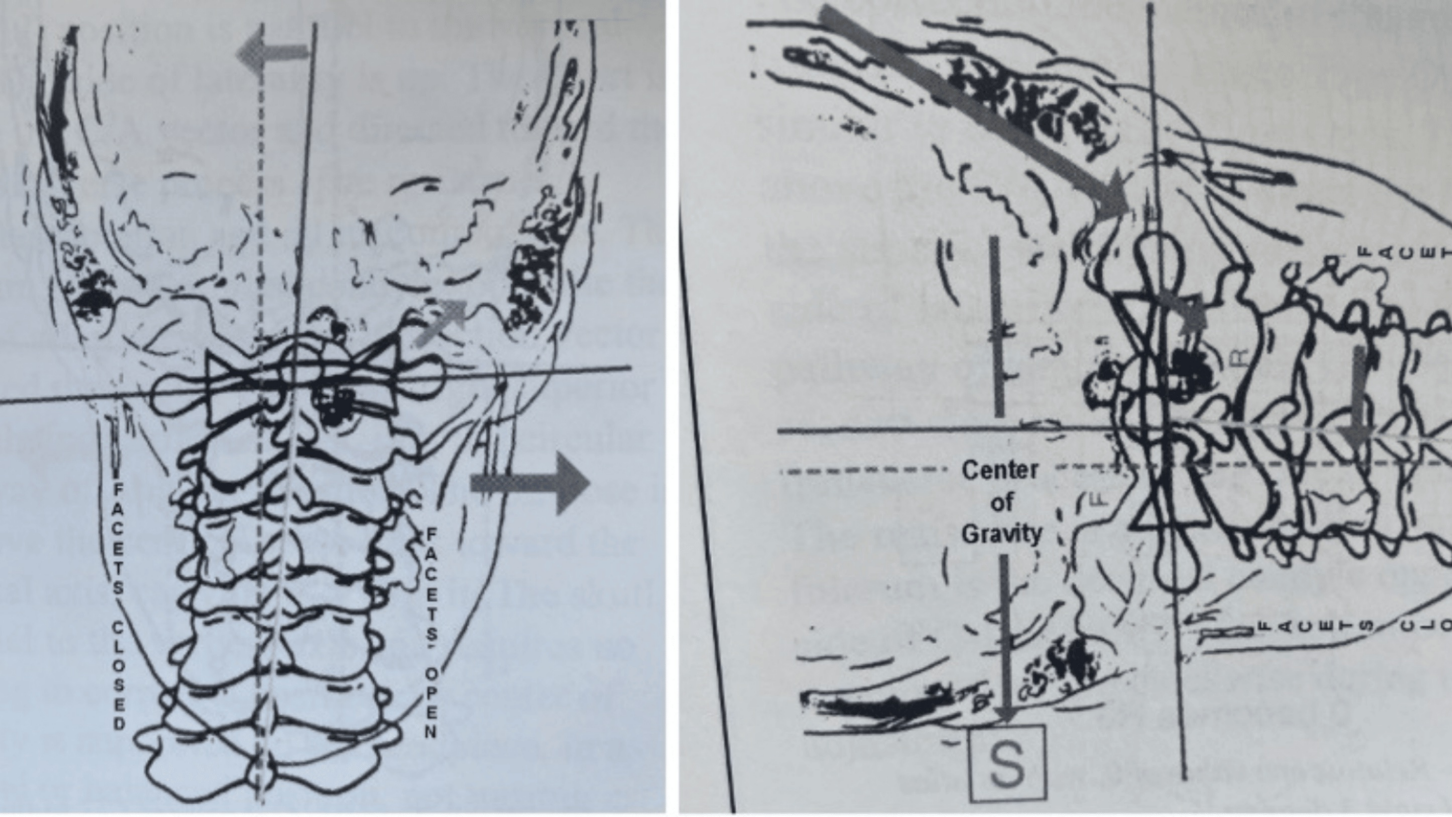
The basic type 1 misalignment pattern (left) and the factors needed to correct the alignment (right) Courtesy of and published in *NUCCA Protocols and Perspectives: A Textbook for the National Upper Cervical Chiropractic Association* [16]. Permission was obtained for use in this article.

One of the most significant expansions of NUCCA protocols, further developed by Dr Ralph Gregory, was the adjustment refinement broken down into several phases (Figure 5) [21]. NUCCA protocol was also defined by four major types of misalignment presentations. It enhanced the understanding of spinal biomechanics, from how the spine compensates for injury and added to the essential factors needed to correct the misalignment optimally (Figure 6) [22]. In 1971, NUCCA formed a research organization to scientifically scrutinize and advance the NUCCA procedure instead of relying on blind faith and philosophy, which was common at the time. Since then, the National Upper Cervical Chiropractic Research Association (NUCCRA), now known as the Upper Cervical Research Foundation (UCRF), has studied and published findings on how the ASC impacts human health and how to optimize the NUCCA procedure [23].

The first mention of chiropractic being used for dizziness intervention was in 1906 when Palmer published, "vertigo [is] the result of deranged nerves” [24]. Over 100 years later, NUCCA practitioners continue to care for patients with dizziness. Advertisements and online discussions regularly host NUCCA chiropractors suggesting their care is effective for dizziness from Ménière's disease (MD). However, as health care and health sciences continue to move towards an evidence-informed model, we must examine the available evidence and identify gaps. This narrative review shall accomplish the following: 1) synthesize the available evidence that the ASC may generate dizziness; 2) synthesize the available evidence on NUCCA intervention’s effect on dizziness; 3) report on gaps in the knowledge base to guide future research.

## Review

Methods

Databases and Inclusion Criteria

Index to Chiropractic Literature (ICL), PubMed®, and Cochrane Library of Systematic Reviews (CLSR) databases were searched using the terms “NUCCA AND Dizziness”, “Atlas Subluxation AND Dizziness”, “Upper Cervical Spine AND Dizziness”, “Craniocervical Junction AND Dizziness”, and “Chiropractic AND Meniere’s” from 2014 through July 2024. Articles selected for the synthesis met one or more of the following inclusion criteria: (1a) specifies using upper cervical technique (UCT) for the ASC; (1b) human subject(s); (1c) diagnosed with "dizziness"; or (2) basic science research on the ASC affecting vestibular anatomy. Articles that used spinal manipulation, or any other form of manual therapy that is not UCT, were excluded. The search results are found in Table 2.

**Table 2:** Literature review search results ICL: Index to Chiropractic Literature; NUCCA: National Upper Cervical Chiropractic Association; CLSR: Cochrane Library of Systematic Reviews

Database	Search Terms	Number of Hits	Number of Papers Meeting Criteria
ICL	NUCCA AND Dizziness	3	3
ICL	Atlas Subluxation AND Dizziness	9	7
ICL	Upper Cervical Spine AND Dizziness	8	5
ICL	Craniocervical Junction AND Dizziness	1	1
ICL	Chiropractic AND Meniere’s	11	8
PubMed®	NUCCA AND Dizziness	0	0
PubMed®	Atlas Subluxation AND Dizziness	4	0
PubMed®	Upper Cervical Spine AND Dizziness	49	0
PubMed®	Craniocervical Junction AND Dizziness	15	0
PubMed®	Chiropractic AND Meniere’s	2	0
CLSR	NUCCA AND Dizziness	0	0
CLSR	Atlas Subluxation AND Dizziness	0	0
CLSR	Upper Cervical Spine AND Dizziness	22	0
CLSR	Craniocervical Junction AND Dizziness	0	0
CLSR	Chiropractic AND Meniere’s	0	0
TOTAL	Not Applicable	124	24

Discussion

Literature Synthesis

124 total papers were identified. 24 papers matched the inclusion criteria for synthesis by clearly identifying ASC as a clinical entity in a patient that was diagnosed with dizziness and who underwent upper cervical low-force (UCLF) care. Of these papers, 10 appeared in multiple searches, leaving 14 original articles. All 14 articles were case studies or series reporting on 20 total patients (Table 3). Taken together, this collection of case studies comprises a cohort of dizziness patients who harbored ASC, and who experienced improvement or resolution of their symptoms after UCLF care. No experimental studies such as clinical trials, basic science studies, or review articles were found in this search, leaving the results susceptible to bias and placebo effects. Of the 14 case studies/series, three were specific to NUCCA UCT, while 11 utilized a different UCT as their intervention. Of the 14 case studies, the dizziness-generating diagnoses were as follows: five involved concussion or other physical trauma to the CCJ, seven involved MD, one involved primary CNS tumor, and one involved dysautonomia to include low heart rate. Listed below are multiple conditions found within the literature review and are discussed in further detail how the ASC is relevant to each condition, how NUCCA intervention may improve outcomes, and further research recommendations.

**Table 3:** Summary of literature review [25-38] NUCCA: National Upper Cervical Chiropractic Association; MD: Ménière's disease; HIO: Hole-in-one

Author	Date Published	UCT Used	Diagnosis	Study Design	Comments
Berner, Steward	03/14/2020	Orthospinology	MD	Case report	68-yo female with self-reported symptom improvement
Chung	08/31/2019	NUCCA	Post-concussion syndrome	Case series of two	16-yo female and 30-yo female with self-reported symptom improvement
Moore	02/11/2019	NUCCA	Post-concussion syndrome	Case series of six	Age ranges from 39 to 82 with objective outcome measure improvement
Finn, Ierano, Doyle	06/16/2022	Atlas Orthogonal	Axial trauma	Case report	49-yo female with subjective improvement and self-reported quality of life improvement
Burcon	05/10/2021	HIO	MD and concussion	Case report	51-yo female with subjective improvement stating her vertigo went from a 10 to a 1
Osborne, Rauch	01/27/2021	Atlas Orthogonal	Dysautonomia	Case report	74-yo female with objective heart rate variability changes and subjective improvements
Null, Null	03/04/2019	Grostic	Traumatic brain injury	Case report	19-yo male with self-reported improvement
Ball	07/03/2017	HIO	Primary CNS tumor	Case report	Unspecified age and gender with objective outcome measurement improvement
Burcon	03/21/2023	HIO	MD	Case report	48-yo female with self-reported symptom resolution of all complaints except hearing loss
Belcher, Barnes	12/19/2022	Atlas Orthogonal	MD	Case report	59-yo female with self-reported resolution of vertigo
Malachowski, Britt	08/17/2020	Knee Chest Upper Cervical Specific	MD	Case report	64-yo female with self-reported improvement
Grey, Ellis	09/30/2019	Blair	MD	Case report	59-yo female with positive improvement
Chung, O’Connell	01/09/2017	NUCCA	MD	Case report	45-yo male with self-reported quality of life improvement and symptom resolution
Pennington, Miller	11/30/2015	Atlas Orthogonal	MD	Case report	63-yo female with reported symptom reduction

ASC as a Cause or Contribution to Dizziness: Pathophysiology

Four mechanistic rationales exist in the literature to explain how the ASC can cause or contribute to the generation of dizziness. The four, described below, are: proprioceptive dysafferentation, craniospinal hydrodynamic dysfunction, direct mechanical irritation from spinal cord tension, and venous compression from spinal cord tension. 

Proprioceptive dysafferentation: Also referred to as somatosensory input hypothesis, proprioceptive dysafferentation holds the highest level of biological plausability and most significant scientific community acceptance regarding dizziness resulting from cervical soft tissue according to the Bárány Society [39]. Mechanoreceptors are a group of proprioceptors within the sensory portion of the nervous system [40]. Their function is to relay tactile sensations and position sense to the neuroaxis. The muscle spindle mechanoreceptors are located in musculoskeletal tissue and respond to length and stretch changes. Muscle spindles are in great abundance within the suboccipital muscles [41,42]. A positional change of only 0.4º in the upper cervical spine is sufficient to cause an increase in afferent discharge [43]. The central vestibular system relies on a constant sensory flow of peripheral information for processing spatial awareness. At rest, the healthy and normal functioning visual and vestibular labyrinth organs relay their typical afferents. However, if the ASC is present, it is hypothesized proprioceptive afferents will increase, resulting in a sensory mismatch and dizziness. 

Craniospinal hydrodynamic dysfunction: A new area of investigation into how the ASC can cause neurological dysfunction, and ultimately dizziness, is through hydrodynamics [11,44-46]. The CCJ is an essential and sensitive anatomical convergence for all fluids moving to and from the brain. An uninterrupted inward flow of arterial blood, outward flow of venous blood, lymphatic movements, and dual movement of cerebral spinal fluid must occur for proper brain and nerve function [10,11]. An obstruction to the arterial blood to the brain is one of the leading causes of death worldwide [47]. However, a non-obstructed, functional reduction of arterial flow can result in nervous dysfunction, such as in cerebral hypoperfusion from orthostatic hypotension, which is known to cause dizziness [7,48,49]. Flammer researched and reported on non-obstructive, functional hypoperfusion of the optic nerve resulting in visual acuity problems, which could also be expected to result in the experience of dizziness [50]. Since the vertebral artery makes four 90º turns within the CCJ and traverses through the atlas transverse processes, it is susceptible to obstruction of blood flow from bony malposition; 20% of cervical rotation and extension is enough to limit blood flow [51-54]. A transverse misalignment of the atlas (rotated atlas) over the axis would potentially reduce the amount of cervical rotation and extension needed to restrict the lumen. The vertebral artery eventually branches off to supply nutrients to the vestibular nuclei [55]. A misalignment of the CCJ may ultimately lead to hypoperfusion of the vestibular nuclei, resulting in dizziness. A clinical component of Wallenberg syndrome, which is a blockage of the posterior inferior cerebellar artery (PICA) that supplies blood to the central vestibular anatomy, is vertigo [52]. Therefore, hypoperfusion, but not complete blockage, is hypothesized to result in vertigo/dizziness as well. Since the CSF has cranial-caudal movement through the C0, C1, and C2 vertebrae of the CCJ, the flow pattern is also susceptible to bony misalignment, particularly from the dentate-ligament spinal cord-distortion hypothesis, as discussed in the next section [11,56]. The CSF traverses the spine and enters the cranium, in a cranial direction, by way of the subarachnoid space. It moves out of the cranium in a caudal direction from the fourth ventricle through the central canal of the spinal cord. Since the spinal cord has direct soft tissue attachment to the bony segments of the CCJ (see the dentate-ligament spinal cord-distortion hypothesis), a structural misalignment is hypothesized to disrupt the normal CSF flow through the CCJ [11].

Improper flow of the CSF and glymphatic pathways can result in waste buildup and reduced nerve function, including the central vestibular processing anatomy [57]. Venous outflow from the cranium can also be altered by jugular vein compression by the transverse process of the atlas [58]. Dysfunctional craniospinal hydrodynamics has been associated with many neurological conditions such as Parkinson’s (known to lead to orthostatic hypotension and dizziness), multiple sclerosis (MS) (known to lead to dizziness and imbalance), and dementia [59-73]. In fact, an MRI study on MS patients with prior craniocervical trauma found that their upright CSF flow and pressure gradients had significant obstruction [74]. Only further research will help us determine if it plays a role in vestibular pathophysiology.

Spinal cord tension and direct mechanical irritation and venous compression: One of the original hypotheses on how the ASC can result in distal bodily dysfunction is the dentate-ligament spinal cord-distortion hypothesis presented by John D. Grostic Jr [56]. This hypothesis presents two mechanisms: direct mechanical irritation of nerves and venous compression. The spinal cord is directly attached to the foramen magnum, the second and third cervical vertebrae, the posterior longitudinal ligament, rectus capitus posterior minor muscle, and through dural attachment to the periosteum of the atlas [75]. Between the attachments of the nerve roots exist bands of tissue called the dentate ligaments. These ligaments are incredibly strong and have their peak strength within the upper cervical spine [76].

The dorsal spinocerebellar tract (DSCT) ascends on the lateral portion of the spinal cord. The DSCT relays proprioceptive afferents from the periphery to the ipsilateral cerebellum. Due to the DSCT’s anatomical location, it is the most vulnerable to mechanical irritation from inappropriate tension applied by the dentate ligaments, for example from ASC. Mechanical irritation of the DSCT is hypothesized to result in dysafferentation, as described above, which may result in sensations of imbalance. The small veins of the upper cervical spinal cord are scarce compared to other regions of the spine. Mechanical obstruction of these veins can cause stasis of blood and local ischemia. These veins move blood at a very low pressure, predisposing them to easy occlusion by compressive forces [56,76]. Gillilan stated that the dentate ligaments may be a means of transmitting mechanical stress to the cord and resulting in small vein occlusion [77]. Local ischemia by means of venous compression and direct mechanical tension on the DSCT are two theories as to how a CCJ misalignment may affect the normal proprioceptive afferents through the dentate ligaments, causing spinal cord tension. The DSCT carries proprioceptive afferents from the periphery to the cerebellum for higher-order processing. The cerebellum corrects posture based on the information it receives from the DSCT. If the information is faulty due to the mechanism(s) explained above, postural abnormalities (and postural symptoms) may result. This hypothesis also adds fuel to the two previous hypotheses, as cord distortion may result in altered craniospinal hydrodynamics and proprioceptive dysafferentation. 

ASC and MD

MD is a vestibular disorder characterized by spontaneous episodes of vertigo, sensorineural hearing loss, and fluctuating aural symptoms [78]. Historically, MD is associated with over-accumulating endolymph in the semicircular canals and otoliths. The most accepted pathophysiology is that this over-accumulation, termed endolymphatic hydrops (EH), damages cochlear anatomy and over-stimulates the peripheral vestibular organs, leading to a unilateral vestibular imbalance, resulting in the symptoms mentioned above [79]. However, recent evidence suggests that EH alone is not enough to cause MD [80]. Endolymph is created by the secretory cells in the vascularis of the cochlea and the dark cells of the labyrinth, which moves to the semicircular canals. It is drained through the endolymphatic duct into the endolymphatic sac. There it extends through the distal vestibular aqueduct and out the external aperture of the aqueduct, where it ends in the epidural space of the posterior cranial fossa. The endolymph has a high K+:Na+ ratio, resembling intra-cellular fluid, as opposed to the perilymph, which has a high Na+:K+ ratio. The perilymph bathes the exterior portion of the membranous labyrinth [81-84]. Not present in the primary literature search because it is a newer publication and not yet indexed is Steward’s retrospective case series on UCT and vestibular rehabilitation on eight patients with vestibular diagnoses [85]. The author measured dizziness handicap inventory (DHI) scores before and after a 30-day plan of care. Of the eight participants, four of them were diagnosed with MD. Their DHI improvement was 64% (Case 1), 28% (Case 2), 92% (Case 3), and 78% (Case 4). It is worth noting that Cases 1-3 received an individual plan of vestibular rehabilitation in addition to UCT, while Case 4 only received UCT. As listed, seven other case reports were found in the literature on UCT and MD's improvement. Steward’s paper noted an objective reduction of the CCJ misalignment, as visible on X-ray, after the UCLF intervention in all cases.

Also not present in the primary search was Burcon’s paper, which retrospectively reported on the health outcomes of 300 MD patients who underwent UCT care [86]. Of these 300 patients, the average pre-care reported vertigo severity on a scale of 1-10 (10 being the worst) was 8.5. At six weeks post care, the average lowered to 3. In one year, it reduced to 2. At six years, it dropped to 0.8. A notable reported cohort feature was that 100% of the cases had a history of whiplash trauma (cervical acceleration-deceleration injury). Though the pathogenesis and pathophysiology of MD is still not fully established, we can use contemporary knowledge of vestibular anatomy and physiology to understand what is being affected. Since vertigo is the main vestibular symptom, we can reduce the generating anatomy to a unilateral imbalance of the semicircular canals, the central processing anatomy of the canals, and/or the neurological pathway between the two [78,81,82]. Therefore, further research on the ASC’s association with MD pathophysiology should focus on the intimate connection between the CCJ and this anatomy. Disrupted hydrodynamics of the CSF with a subsequent restriction of endolymph movement due to CCJ misalignment should be evaluated. 

ASC and Vestibular Migraine

Vestibular migraine is a migraine disorder that accompanies the vestibular system. It is estimated that it may affect up to 1% of the general population. Though no evidence was found in the primary literature search on UCT and vestibular migraine, a clinical trial on NUCCA intervention on non-vestibular migraine has been published [87]. The study found that NUCCA intervention significantly increased neurologist-measured quality of life measures and had some improved influence on blood and CSF flow patterns post treatment. No adverse events were reported. 

ASC and Concussion

It is estimated and theorized that the cervical spine ligaments are injured in every single concussion due to the force and cervical torsion that it takes for a concussion to occur [88-90]. Since the CCJ is the most mobile unit of the cervical spine, it is the least stable, allowing for ligamentous damage, structural misalignment, and ultimately for the ASC to form [91]. Post-concussion syndrome occurs when symptoms last beyond three months [92]. A common symptom of post-concussion syndrome is dizziness. ASC and cervical spine-related dizziness research is complicated when studying concussions due to the vestibular anatomy typically being injured during a concussion. However, Moore’s paper on using NUCCA to intervene with post-concussion patients found objective clinical improvement with many symptoms, including dizziness [27]. Moore also noted an objective reduction of the structural misalignment, as measured on X-ray, after the UCLF intervention in all cases.

ASC and Autonomic Function

A pilot, placebo-controlled, randomized clinical trial assessed NUCCA intervention on hypertension outcomes [93]. The study concluded, “Restoration of Atlas alignment is associated with marked and sustained reduction in BP similar to the use of two-drug combination therapy” [93]. The mechanisms of how this was possible were discussed in the craniospinal hydrodynamic dysfunction section above. Since this study found that realignment of the ASC achieved ideal blood pressure, it would be wise to follow up this study in postural orthostatic tachycardia syndrome and orthostatic hypotension patients to assess for improvements in lightheadedness, dizziness, and improved orthostatic blood pressure.

Cervical Dizziness (CD)

In the Bárány Society’s consensus paper on CD, appropriate terminology is discussed. They state, “The aetiology is unclear, or at least the data to support underlying mechanisms in humans are inconclusive, and there is no diagnostic test, the term Cervicogenic implies a mechanistic knowledge that is currently lacking. Hence, we propose the term Cervical” [39]. Since our paper discusses the ASC as a specific etiology of dizziness, we have decided not to use the terms "cervical dizziness" or "cervicogenic dizziness" for discussion.

Acute Cerebral Vascular Ischemia and BPPV

As discussed in this review, many conditions can generate dizziness. Saying this, there are some that UCT intervention should not be studied for efficacy. Firstly, acute cerebral vascular ischemia, such as a stroke. A stroke is an acute life-threatening emergency that should only be treated in the emergency medicine setting [94]. Secondly, classic BPPV. Accurately diagnosed BPPV has nearly a 100% cure rate with the appropriately administered canalith repositioning maneuver [3].

ASC Without Dizziness

Studies exist that document patients presenting with ASC, yet they do not have dizziness [87,93]. A fair question is, why doesn’t every patient who presents with an ASC also have dizziness? Since four theories were reported as to how the ASC can generate dizziness, it may be a combination of the pathogenic mechanisms plus genetic predisposition, previous vestibular trauma, demographic factors, and lifestyle, amongst many other variables that are required. In other words, the ASC might be a contributor to dizziness generation rather than a cause. Studies must be conducted to answer this question without speculation.

Future Research

While healthcare and health sciences continue to move towards an evidence-informed model, resources must be allocated to conducting gold-standard, high-quality, placebo-controlled, statistically measured experimental research. Since the NUCCA intervention uses very minimal force, most patients report that they are unaware that an intervention took place during care. This puts NUCCA in a strong position to remove participant bias by implementing a placebo-control in clinical trial research, such as used in the NUCCA hypertension study [93]. This review has outlined four gaps in the literature and expresses a need for the following: Clinical trials: Well-designed, blinded, randomized, and placebo-controlled clinical trial research should be conducted to examine NUCCA’s effect on specific vestibular conditions. Since vestibular diagnosis has historically been weak, every attempt should be made to accurately include only participants that meet the current and specific diagnostic criteria per inclusion of each study. This may include collaborating with specialist such as neurotologists and radiologists. In addition to objective dizziness changes, the studies should also assess quality of life measures. A foundational starting point may be to form a practice-based research network (PBRN) and compile data on many different vestibular diagnoses and how they improve under NUCCA care. Clinical trials should then be conducted on the diagnoses that responded the most favorably during the PBRN. This strategy will help focus resources and save on research costs. Studies must follow the NUCCA protocol closely and observe the Restoration Principle to accurately assess the intervention’s effect [8].

MD: A further and deeper exploration of the ASC’s possible contribution to MD pathophysiology. Future studies should examine the relationship between the CSF and the endolymph and whether the ASC affects endolymph hydrodynamics. Also, studies should assess for a proprioceptive contribution from the ASC to MD pathophysiology. Recent advances in MRI technology have allowed the endolymph to be viewed, which is helpful in future studies [95].

The ASC's affect on vestibular anatomy: To better understand the neurophysiological changes from the ASC, suboccipital muscle mechanoreceptor afferent discharge should be compared to individuals presenting with ASC versus a normal group. Sensorimotor testing may be valuable to determine differences between the ASC and normal groups. Further and deeper exploration of how the ASC can have negative consequences and imbalances in relation to the peripheral vestibular organs and the central vestibular processing anatomy is warranted. For example, testing if the ASC has direct negative sensory consequences to the vestibular semicircular canals.

The ASC's affect on craniospinal hydrodynamics: Though Flanagan's paper examined how the CCJ can be a "choke point" for craniospinal fluid movement, it did not directly examine the ASC as a cause [11]. Woodfield et al. found improved cerebral spinal fluid flow patterns post-NUCCA intervention of the ASC in migraine cases, laying a solid foundation to follow up this study for a better understanding of hydrodynamics as they relate to the ASC [86]. 

## Conclusions

The present review describes multiple theoretical mechanisms by which ASC-evoked dizziness may be mitigated by UCLF intervention, and presents a literature synthesis with observational evidence suggesting that further studies (randomized controlled trials (RCTs)) are warranted to explore the relationship between upper cervical chiropractic care and resolution of dizziness symptoms. Such studies may inform clinicians of novel intervention strategies for patients presenting with complaints of dizziness, thereby augmenting a current deficit in success of treatment of this symptom.
